# Automated analysis of pectoralis major thickness in pec-fly exercises: evolving from manual measurement to deep learning techniques

**DOI:** 10.1186/s42492-024-00159-6

**Published:** 2024-04-16

**Authors:** Shangyu Cai, Yongsheng Lin, Haoxin Chen, Zihao Huang, Yongjin Zhou, Yongping Zheng

**Affiliations:** 1 School of Biomedical Engineering, Medical School, Shenzhen University, Shenzhen, 518073 China; 2 Department of Biomedical Engineering, the Hong Kong Polytechnic University, Hong Kong, 999077 China

**Keywords:** B-mode ultrasound, Deep learning, Exercise training, Pectoralis major, Wearable ultrasound-imaging biofeedback

## Abstract

This study addresses a limitation of prior research on pectoralis major (PMaj) thickness changes during the pectoralis fly exercise using a wearable ultrasound imaging setup. Although previous studies used manual measurement and subjective evaluation, it is important to acknowledge the subsequent limitations of automating widespread applications. We then employed a deep learning model for image segmentation and automated measurement to solve the problem and study the additional quantitative supplementary information that could be provided. Our results revealed increased PMaj thickness changes in the coronal plane within the probe detection region when real-time ultrasound imaging (RUSI) visual biofeedback was incorporated, regardless of load intensity (50% or 80% of one-repetition maximum). Additionally, participants showed uniform thickness changes in the PMaj in response to enhanced RUSI biofeedback. Notably, the differences in PMaj thickness changes between load intensities were reduced by RUSI biofeedback, suggesting altered muscle activation strategies. We identified the optimal measurement location for the maximal PMaj thickness close to the rib end and emphasized the lightweight applicability of our model for fitness training and muscle assessment. Further studies can refine load intensities, investigate diverse parameters, and employ different network models to enhance accuracy. This study contributes to our understanding of the effects of muscle physiology and exercise training.

## Introduction

Skeletal muscle training is crucial for enhancing athletic performance and the overall quality of life [1–4]. Real-time visual biofeedback, particularly real-time ultrasound imaging (RUSI), has made significant progress in sports medicine and rehabilitation. It provides non-invasive, instantaneous, and detailed insights into the human body [5–7]. RUSI, a type of visual biofeedback, is exceptionally useful for real-time monitoring of skeletal muscles [8–10]. For example, Henry and Westervelt [6] demonstrated in their research that abdominal contraction training is more effective when supplemented with RUSI visual biofeedback.

**Fig. 1:**
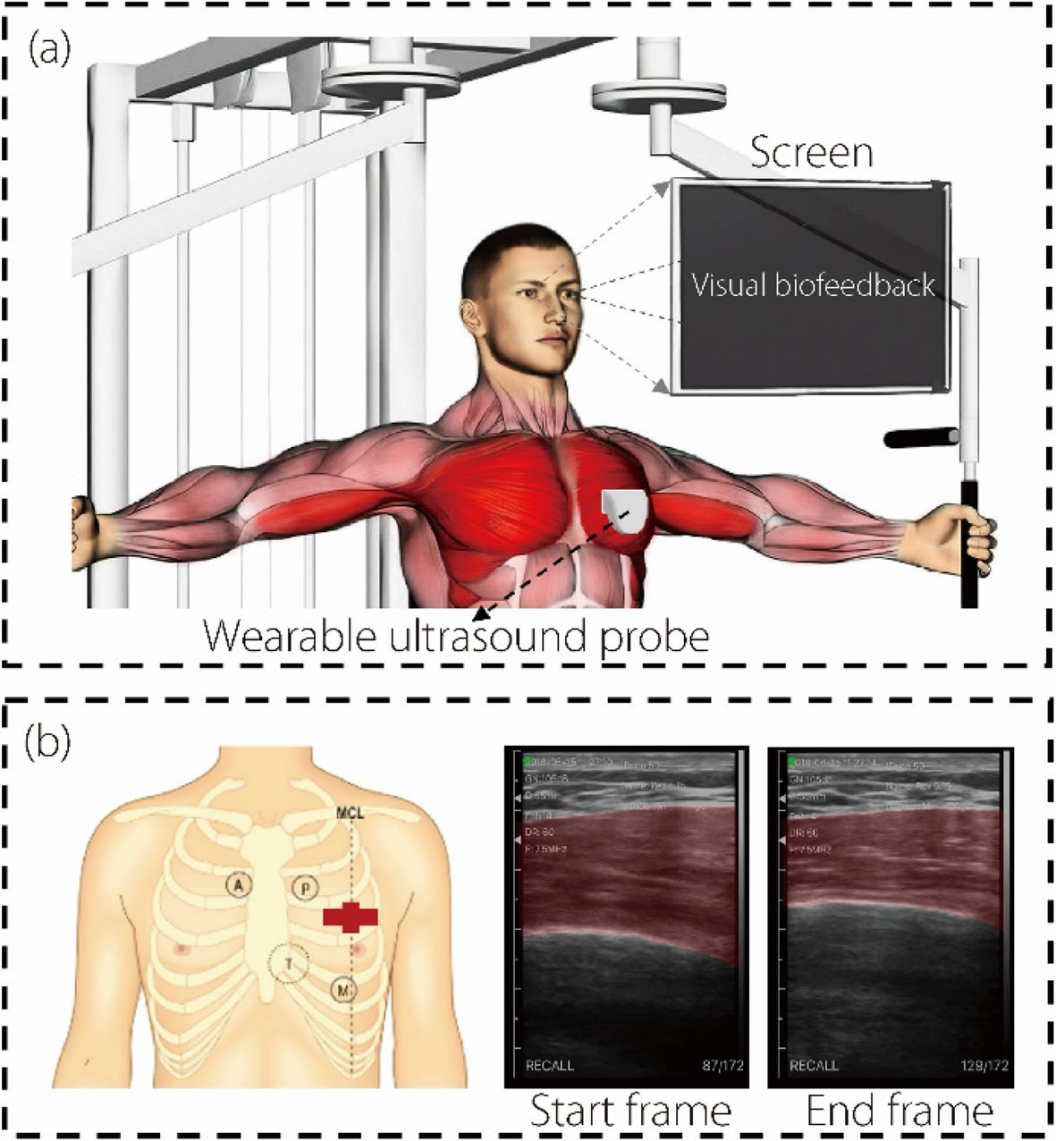
Experiment setup for probe location and data processing. **a** Schematic diagram of the experiment [11]; **b** Left, proposed probe placement in the anatomy: the red cross indicates the probe location. Right, representative ultrasound frames of PMaj, two frames represent the start frame and end frame (scanning depth is 55 mm and each small segment is 5 mm). The ground truth with manually labeled highlights

Our team recently developed a portable ultrasonography-based RUSI visual biofeedback system for pectoralis major (PMaj) exercises (pec-fly) in a cohort of 25 athletes [9]. The system significantly enhanced the PMaj exercise effectiveness, as revealed by the RUSI probe in the area between the third intercostal space and the midclavicular line on the left side (Fig. 1a). However, the thickness measurement procedure still involves manual operations [9], such as manually selecting both the location and line segment for thickness determination.

Advancements in deep learning have improved the automatic extraction of tissue contours using ultrasound imagery. Examples include the UNet model proposed by Ronneberger et al. [12], breast ultrasound image segmentation with an extended UNet architecture proposed by Guo et al. [13], multiscale feature-aggregated UNet for intravascular ultrasound image segmentation by Xia et al. [14], and recent advancements in ultrasound image segmentation using the transformer model [15–18]. Despite recent developments, the UNet model is still preferred because of its lightweight deployment capabilities [19].

This study aimed to develop a portable and lightweight ultrasound-based RUSI biofeedback system. To achieve this, we propose using a deep learning model for frame-by-frame segmentation of the ultrasound image stream. This method promises automated and precise analysis of PMaj thickness variations, offering a detailed view of changes in the coronal plane that could improve the intelligence and efficiency of current systems [9]. In addition, this study can clarify or confirm certain phenomena in biomedical research by examining variations in PMaj thickness.

The remainder of this paper is organized as follows. Methods section  firstly outlines the material used in this study, including system specifications, diagnostic data, and data volumes; secondly a comprehensive exposition of the deep learning models employed is presented, delineating the motion estimation and image segmentation branches thirdly, we elaborate on our methodology for evaluating the PMaj thickness of segmentation results and discuss the findings therein; finally, we evaluate modifications applied to the model, including disintegration experiments performed on the RNN components and enhancements made to the UNet network. Results section  presents the results, highlighting the model’s optimization and performance evaluations, coupled with comparative analyses of diverse model outcomes. In Discussion section, a comprehensive analysis is conducted to assess the variances in thickness among different condition groups, along with the changes across different measurement locations of the maximum thickness. Finally, we engage in a discussion of our research findings and their potential physiological significance.

## Methods

### Materials

**Fig. 2:**
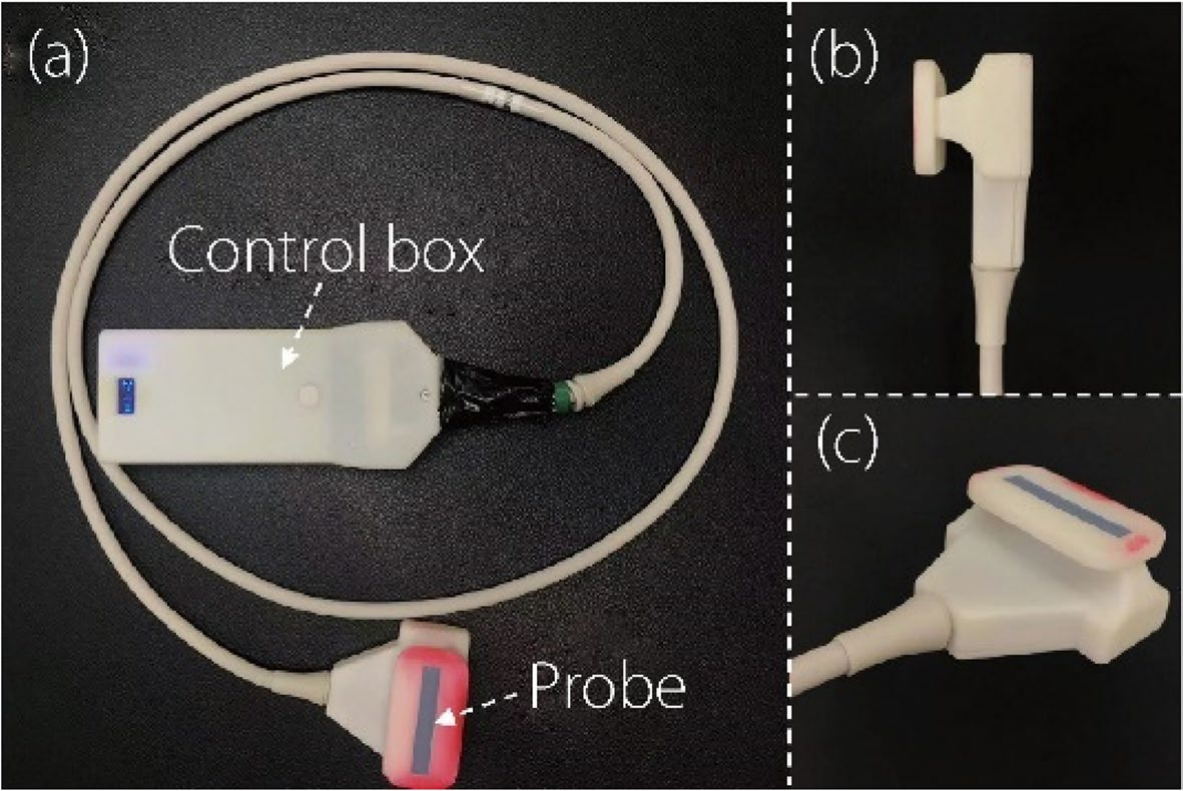
Wearable RUSI biofeedback system: ultrasound image acquisition unit wirelessly connected to the matched smartphone-based application

The RUSI biofeedback system and its training configuration are illustrated in Fig. 2. This system comprises two main components: (a) a bespoke ultrasound image-acquisition unit and (b) a mobile terminal (Fig. 1). Specifically, the image acquisition unit includes a custom-designed ultrasound probe measuring 4.5 cm × 0.7 cm, a signal cable, and a control box with dimensions of 15.6 cm × 6 cm × 2 cm. The weight of the probe is approximately 350 g, making it lightweight and easily maneuverable during operation. Operating at an ultrasound frequency of 7.5 MHz with a 35% bandwidth, the probe is appropriate for superficial muscle imaging based on previous research [8]. Its scanning depth ranges from 20 mm to 55 mm, allowing for versatile applications across various muscle groups and anatomical structures. The control box contains the components for signal processing and data transmission. This unit utilizes 5G Wi-Fi technology for seamless transmission of ultrasound images between the ultrasound probe and the mobile terminal, ensuring a stable connection with a theoretical maximum speed of 450 Mbps. This high-speed connection, coupled with the system’s support for the 802.11 g Wi-Fi standard operating at a 20 MHz frequency, provides reliable data transmission while maintaining compatibility with modern smartphone devices. An application installed on a smartphone displayed these B-mode ultrasound images, with the system achieving 18 frames per second.

The experimental design and participant selection criteria used in our research were based on previous methodologies [9]. We enrolled 25 healthy young men and used a repeated measures design. The inclusion criteria for the study were as follows: individuals without a history of chest, spine, or upper limb surgery; those who have not faced recent orthopedic or neurological issues; participants without significant chronic medical conditions; and subjects with no previous experience in RUSI feedback training. Additionally, the participants were required to refrain from any upper-body resistance training for a minimum of 48 h before the experiment. The subjects were aged from 18 to 35 years. Before the study, the participants were thoroughly informed of the objectives and procedures of the experiment. All participants were required to sign a consent form. This study received ethical approval from the Hong Kong Polytechnic University Ethical Review Board (No. HSEARS 20180418002).

A meticulous image labeling process was performed to enable comprehensive and automated measurement of the PMaj thickness in the coronal plane within the detection area of the probe. This approach effectively overcomes the limitations associated with previous manual measurement methods for the number of processable images and measurement locations. Under the guidance of a medical professional, the labelers used the Labelme image annotation tool [20] to annotate the selected frames from the PMaj ultrasound video. The annotations focused on the contour of the PMaj muscle within the probe detection region in the coronal plane, including the start and end frames (Fig. 1b). The resulting dataset comprised 13,640 images and 1,136 annotations.

#### Proposed model

**Fig. 3:**
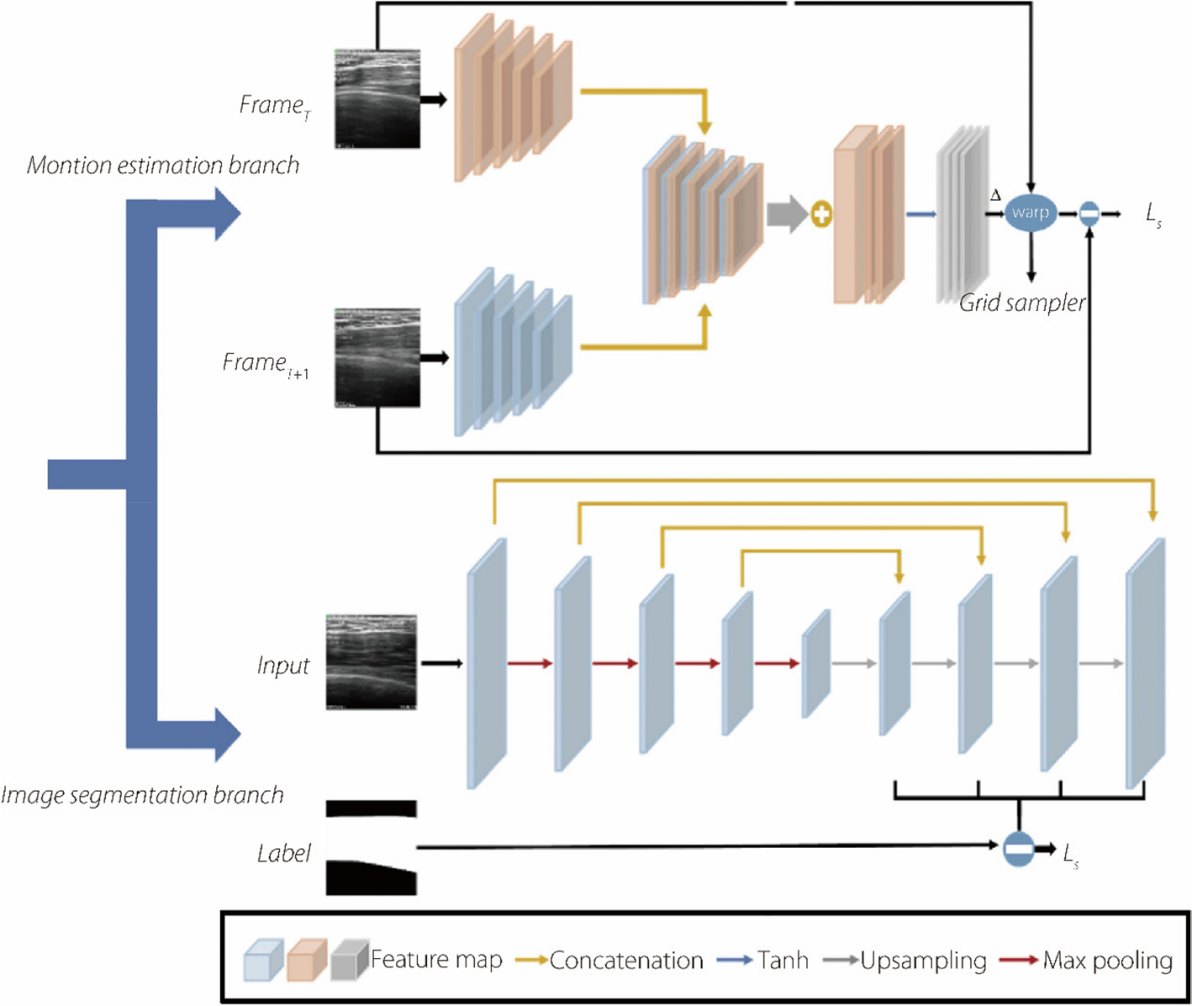
Deep learning models were used in this research. The model is comprised of a motion estimation branch and an image segmentation branch

Our approach utilizes a deep-learning model to measure the PMaj thickness of the processed data (Fig. 3). The model employs a deep learning optical flow motion estimation model to detect motion patterns and applies these findings to motion compensation and artifact removal in medical imaging. In addition, a segmentation model was employed to isolate and analyze features from the designated regions. Our methodology resulted in a joint learning approach that combined the outputs of both the motion estimation and segmentation models. The details of each branch are as follows:

1. The Siamese-style multiscale recurrent motion estimation branch (based on the work proposed by Qin et al. [21]). In our implementation, we replace the recurrent neural network (RNN) [22] component with two convolutional layers.
2. For the segmentation branch, we employed a UNet segmentation network. Unlike the approach proposed by Qin et al. [21], in which the weights were shared, we treated the motion estimation results as the ground truth for segmentation. This is complemented by adopting a deep supervision strategy to ensure the preservation of multiscale information in the segmentation results.

The effectiveness of this integration was measured using the Dice coefficients from Eq. (1) as the key performance metric [21]. To guarantee the stability and reliability of the results, a fivefold cross-validation method was implemented.

1 $$\begin{array}{c}Dice=\frac{\left|Y\cap P\right|}{\left|Y\right|\cup \left|P\right|}\end{array}$$

where *Y* denotes the ground truth and *P* denotes the model output.

#### Methodology for PMaj thickness measurement

**Fig. 4:**
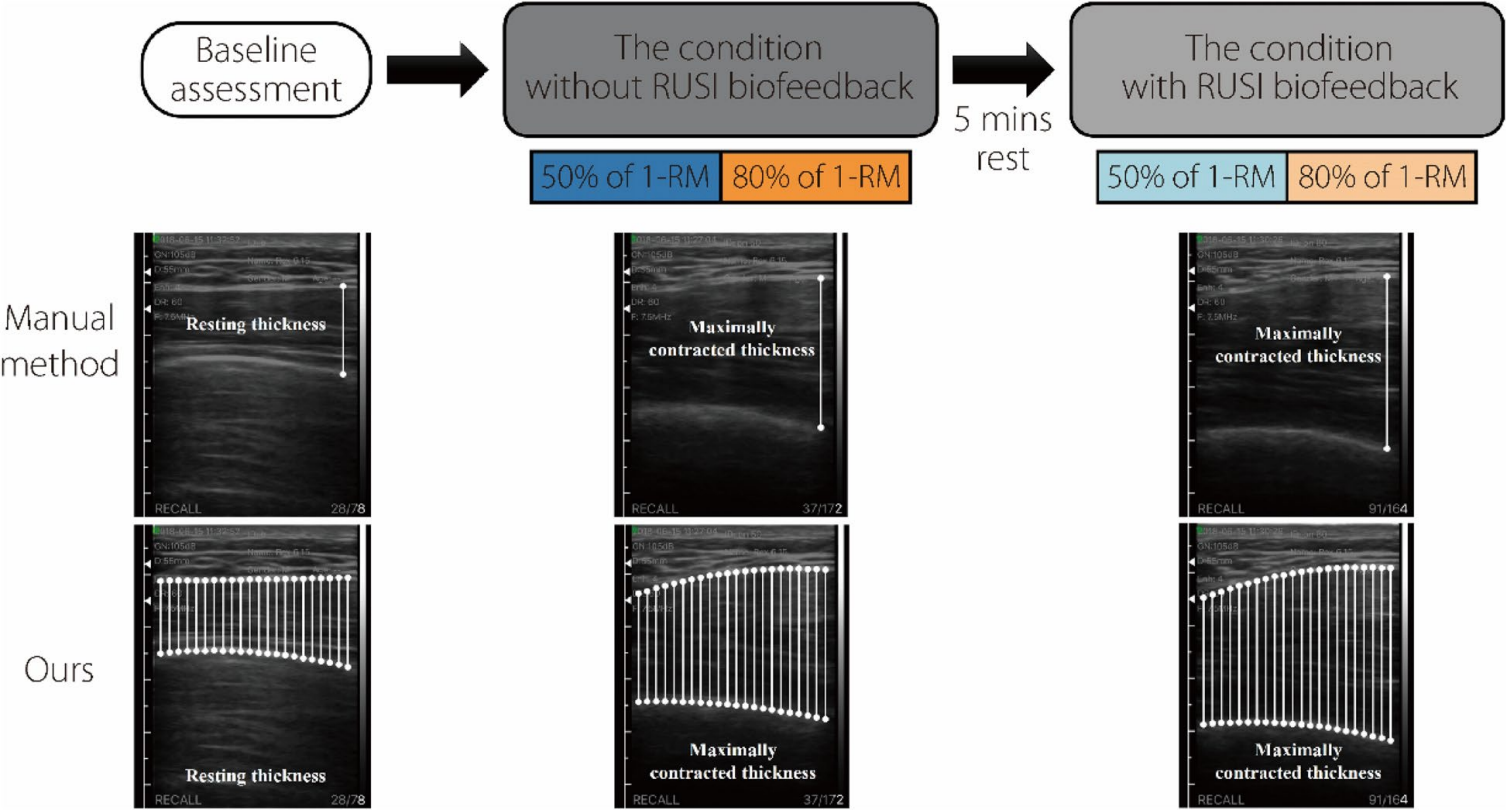
Method outline: Participants were instructed to remain still for approximately 30 s to establish a baseline assessment. This was followed by four experimental conditions of PMaj resistance training. The training session consisted of two assigned load training intensities (50% of 1-RM and 80% of 1-RM), with the conditions without RUSI biofeedback continuously being performed before the condition with RUSI biofeedback. One set of three repetitions was performed in each experimental condition. Our methodology employs a comprehensive automated approach for calculating PMaj thickness across the entire probe detection region (scanning depth is 55 mm and each small segment is 5 mm), in contrast to manual measurement methods [9]

The experimental protocol used in this study was based on the methodology established in our previous manual measurement studies [9]. The complete training protocol is illustrated in Fig. 4. Data collection included exercises under varying load conditions, specifically low-moderate and high loads. These values were quantified as 50% and 80% of the one-repetition maximum (1-RM) [23], respectively. For each intensity level, the participants performed the pec-fly exercise twice, once without RUSI biofeedback, and once with RUSI biofeedback. Before the experimental sessions, we recorded the baseline thickness of the PMaj in a relaxed state without any resistance. Our approach differs from traditional methods that visually estimate the PMaj thickness by identifying the point of maximum distance between the inferior border tip and superior border-bottom. Rather, we utilized an automated system to calculate the PMaj thickness throughout the probe detection region. This method enhances the accuracy and comprehensiveness of the measurements. Muscle activation levels were measured using the muscle thickness change (%) [24–28]. This was defined as the percentage difference in PMaj thickness between the resting state and maximum contraction and was calculated using Eq. (2).

2 $$\begin{array}{c}Muscle thickness-change \left(\mathrm{\%}\right)=\\ \frac{Maximally contracted thickness-Resting thickness}{Resting thickness}\times 100\%\end{array}$$

#### Evaluating model modifications: an ablation study

We conducted ablation experiments on the key components of a deep learning model to validate the effectiveness of our modifications. We focused on two main aspects: modifying the RNN component in the motion estimation branch and applying the UNet network in the segmentation branch.

The transition from RNN to convolutional layers: In the motion estimation branch, as detailed in Fig. 3, the original RNN component was replaced with two convolutional layers. We evaluated the impact of this change by comparing the model’s parameter count and performance in both the original and modified states. The performance evaluation focused on the accuracy of the model in capturing motion patterns and compensating for motion for medical image processing.

We employ a UNet network in the segmentation branch, diverging from the weight-sharing scheme proposed by Qin et al. [21]. We used the motion estimation outcomes as ground-truth data for segmentation, incorporating a deep supervision approach to preserve multiscale information in the segmentation results. To establish the superiority of the UNet network, we compared it with UNet++ [29] and the latest EGE-UNet [30] in terms of the parameter count and model performance.

The ablation experiments were designed to precisely quantify the contribution of each modification to the overall model performance. These experiments were designed to validate the effectiveness of the proposed model for measuring PMaj thickness, confirming the stability and reliability of the results using fivefold cross-validation.

## Results

To optimize the model, we replaced the RNN component with a two-layer convolutional neural network (CNN). This modification aimed to assess whether a simpler and more computationally efficient architecture could maintain or enhance the model performance. The comparison results are presented in Table 1. While the two-layer CNN model improved computational efficiency, it showed a minor reduction in motion estimation accuracy. This outcome suggests that while simplification of the model architecture can lead to faster processing times, it may reduce the precision in certain aspects of performance.

**Table 1:** Performance comparison of RNN and two-layer CNN models

	Smooth loss	MSE loss	Model parameter size
RNN (LSTM) [22]	**0.1004 ± 0.0004**	0.0016 ± 0.0004	21,223,410
Two-layer CNN (our)	0.1005 ± 0.0001	**0.0015 ± 0.0002**	**21,210,289**

Model parameter size: the total number of trainable parameters within the model

A comparative analysis of UNet, UNet++ , and EGE-UNet revealed that each model had similar segmentation effectiveness, as reflected by their Dice coefficients. However, variations were noted in their BCEFocalLoss performances, with EGE-UNet scoring the highest, suggesting potential limitations when generalizing new datasets. UNet++ features dense convolutional blocks and deep supervision, enhancing feature extraction and learning accuracy but at the cost of increased parameters. In contrast, while EGE-UNet prioritizes parameter efficiency, it achieves a halved parameter count without a proportional gain in computational speed. Therefore, UNet emerged as a balanced choice for our segmentation requirements because of its optimal blend of complexity and efficiency. Further details are presented in Table 2.

**Table 2:** Comparative analysis of UNet, UNet++ , and EGE-UNet

	Dice coefficient	Segmentation loss	Computational speed
UNet [12]	**0.938 ± 0.033**	0.110 ± 0.046	97.5 ms
UNet++ [29]	0.938 ± 0.041	**0.108 ± 0.035**	102.5 ms
EGE-UNet [30]	0.938 ± 0.047	0.324 ± 0.016	**92.5 ms**

Computational speed: the time taken by each model to process a single image, measured from input to output

In this study, we chose a combination of a two-layer CNN and UNet as the appropriate model. This was to ensure an optimal balance between maintaining a swift response speed and achieving high model performance. The integration of a two-layer CNN with UNet provided a computationally efficient model that captured the detailed features necessary for accurate segmentation. This combination was effective and did not compromise the rapid processing capabilities required for our analysis.

The performance of the tailored model was evaluated. By training the proposed model, we achieved an average Dice coefficient of 0.94 across each fold of the optimal model. We calculated the change in the PMaj thickness within the probe detection region (located at the intersection of the third intercostal space and the midclavicular line on the left side of the body) under various experimental scenarios using Eq. (2). The study included training intensities at low-moderate (50% of 1-RM) and high (80% of 1-RM) loads, each further categorized based on the pec-fly exercise performed with or without RUSI biofeedback. Additionally, we documented the PMaj thickness at rest, which was denoted as the resting group. Line graphs were used to depict the PMaj thickness change trends, providing visualizing the variations across different load intensities and biofeedback conditions (with and without biofeedback) at each specified load intensity.

**Fig. 5:**
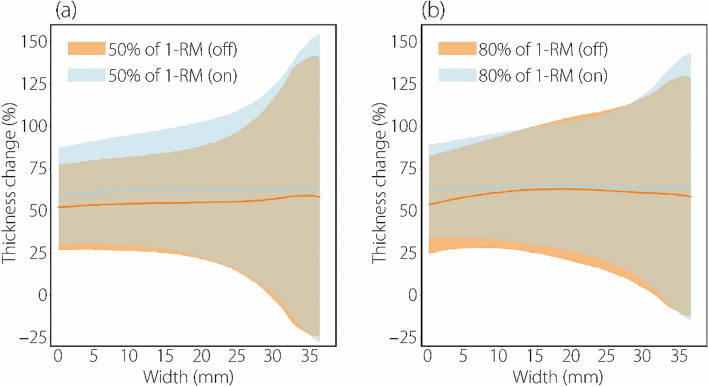
PMaj thickness change for the same load training intensity at two different biofeedback conditions (with and without RUSI biofeedback) (*n* = 25). In graphs, the curves represent the average values of the PMaj thickness change, while the envelope areas represent the standard deviation of the PMaj thickness change. (a) represents the PMaj thickness change with different biofeedback conditions at the low-moderate load training intensity (50% of 1-RM); (b) represents the PMaj thickness change with different biofeedback conditions at the high load training intensity (80% of 1-RM). For the assigned load training intensity with RUSI biofeedback (on), the PMaj thickness change was significantly increased for low-moderate training intensity as well as for high-intensity training compared to the corresponding load training intensity without RUSI biofeedback (off). This emphasizes the important role of RUSI visual biofeedback in the process of muscle thickness change

Previous research [9] demonstrated the effectiveness of RUSI visual biofeedback in enhancing PMaj thickness changes through manual measurements at manually selected locations. Our study expands on this using automated measurements to cover the entire transverse region of the PMaj thickness change in the probe-detection region. This study examined the impact of RUSI visual biofeedback on changes in the thickness of the entire transverse region of the PMaj in the probe detection region at two different training intensities. The results are shown in Fig. 5.

After receiving RUSI biofeedback, the PMaj thickness increased at both training intensities (*n* = 25). Figure 5 shows that the standard deviations of the PMaj thickness change are significantly larger on the right-hand side of the graphs. However, the average values of PMaj thickness change were not significantly different between the two intensities.

**Fig. 6:**
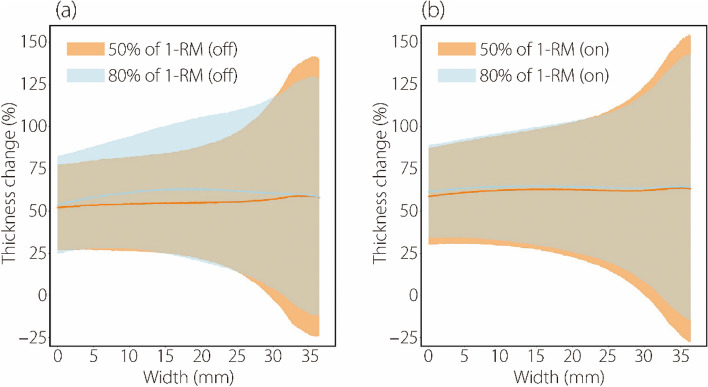
PMaj thickness change for two different load training intensities at the same biofeedback condition (with or without RUSI biofeedback) (*n* = 25). The curves represent the average values of the PMaj thickness change, while the envelope areas represent the standard deviation of the PMaj thickness change. (a) represents PMaj thickness change without RUSI biofeedback at different load training intensities; (b) represents PMaj thickness change with RUSI biofeedback at different load training intensities. It is shown in (a) and (b) that differences in the PMaj thickness change distribution between them

Furthermore, we analyzed PMaj thickness variations at different load training intensities within the same biofeedback condition. The findings presented in Fig. 6 reveal that without RUSI biofeedback, PMaj thickness changes were more pronounced at 80% 1-RM than at 50% 1-RM, particularly in the middle part of Fig. 6a, with less variation at the ends. In contrast, with RUSI biofeedback, the differences in PMaj thickness changes between the two intensities were less distinct, as shown in Fig. 6b. In other words, the data distribution in Fig. 6b does not exhibit the corresponding characteristics (larger differences in the middle part of the graph and smaller differences at both ends) in Fig. 6a.

**Fig. 7:**
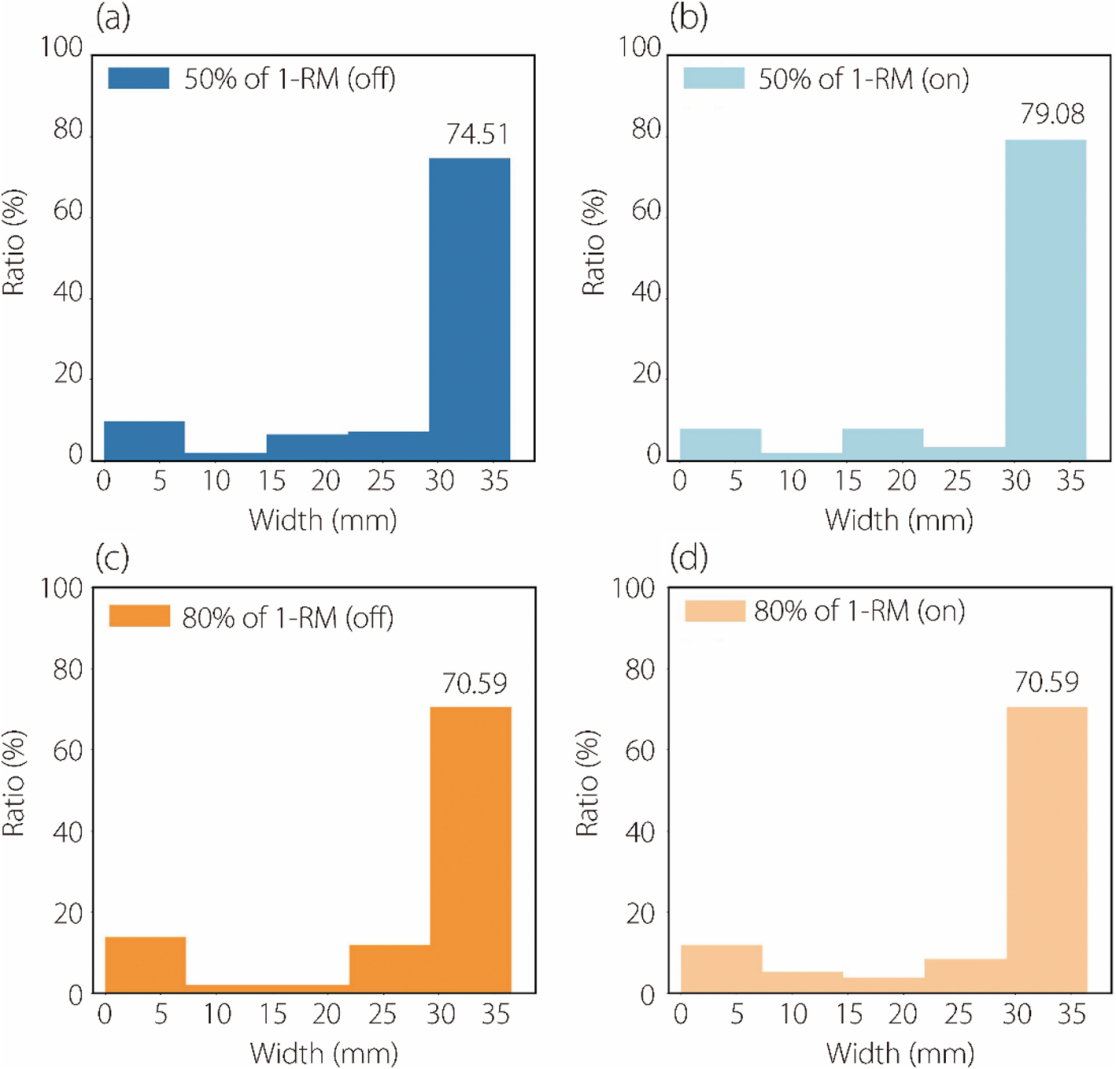
Distribution of the measurement locations of the PMaj maximum thickness change for different experimental conditions (*n* = 25). (a) represents the 50% of 1-RM without RUSI biofeedback; (b) represents the 50% of 1-RM with RUSI biofeedback; (c) represents the 80% of 1-RM without RUSI biofeedback; (d) represents the 80% of 1-RM with RUSI biofeedback. We found that the PMaj maximum thickness occurrences had the highest ratio within the rightmost interval of the graph

Finally, we review the data analysis of a previous study on the selection and measurement of the maximum thickness of the PMaj. To objectively determine the optimal site for measuring peak PMaj thickness, our goal was to pinpoint the location where PMaj thickness is at its maximum. We revisited the data analysis method used in a previous study to determine the maximum thickness change in PMaj under different experimental conditions [9]. Considering the potential impact of boundary effects during image processing [31] that may result from overly narrow spacing, we subdivided the region into five equidistant intervals as much as possible, as illustrated in Fig. 7.

The analysis shows that the maximum thickness change in PMaj was mostly located in the rightmost interval of Fig. 7 under various experimental conditions. This suggests that the measurement locations for the maximal change in PMaj thickness were frequently closer to the rib end on the left side of the body. Differences in experimental procedures or individual participant characteristics could account for variations in PMaj thickness at other locations.

In general, our research confirmed the significant impact of RUSI biofeedback on PMaj exercises based on previous studies [9]. The observations were broadened to cover the entire probe detection area. We discovered that training with wearable RUSI biofeedback devices at 50% and 80% 1-RM loads significantly increased the PMaj thickness compared with training without these devices.

Additionally, we observed notable differences in the PMaj thickness changes between 50% and 80% of the 1-RM loads when conducted without RUSI biofeedback. These variations were less distinct when the RUSI biofeedback was used. Furthermore, the maximum thickness change in PMaj was predominantly observed near the rib end on the left side within the detection area, specifically in the rightmost interval (Fig. 7). These findings highlight the impact of RUSI biofeedback on changes in PMaj thickness and improve our understanding of its role in muscle training. This will contribute to more precise and effective training guidelines for practical applications.

## Discussion

To address the limitations of subjective manual measurements in previous research [9], we utilized a deep learning model to segment the ultrasound images and calculate the PMaj thickness. The segmentation results of the model achieved a DSC of 0.938 ± 0.033, achieving acceptable accuracy and reliability. This method could improve the flexibility and convenience of wearable RUSI systems for real-time monitoring and facilitate a thorough and quantitative analysis of the entire probe detection region.

First, our findings showed an increase in the PMaj thickness in the coronal plane with the incorporation of RUSI visual biofeedback, regardless of training intensity. This aligns with prior research [9] and expands the measurement scope to include the entire probe detection region. After integrating RUSI biofeedback into the pec-fly exercises, we observed adaptive changes in PMaj thickness. The standard deviation was notably higher near the rib end than at the clavicular end (*n* = 25), indicating individual differences in PMaj thickness.

Second, significant variations in the PMaj thickness were observed between 50% and 80% of the 1-RM loads, particularly in the central region, with smaller differences towards the edges. These findings are objective and based on the data collected, without any subjective evaluations. These disparities were considerably reduced by the RUSI biofeedback, indicating a possible alteration in the muscle activation strategies of the participants. This phenomenon, potentially linked to different muscle contraction patterns, has been observed in other muscle training studies [32–34], and it may be linked to different muscle contraction patterns. Further investigation is warranted for the PMaj exercises.

Third, our study aimed to identify the optimal site for measuring the peak PMaj thickness based on prior data [9]. The results showed that the peak PMaj thickness measurements predominantly occurred near the rib end within the probe detection area, suggesting that this is the ideal location for such measurements. This insight could be utilized in future research to accurately determine the peak PMaj thickness measurement locations, particularly near the rib end.

In addition, our findings suggest that the division of training intensities into 50% and 80% 1-RM may have been inadequate, resulting in ambiguous data. Future studies should refine these intensities and collect more data to achieve a more accurate analysis of the RUSI biofeedback effects. Although this requires more data, using automated measurements reduces the additional processing time and cost.

Finally, our model is efficient and can function on a standard personal computer without requiring a GPU [35]. This makes it a practical addition to previously proposed portable ultrasound systems for fitness training [9]. Furthermore, we provided guidelines for accurately selecting measurement locations for the maximum thickness of PMaj within the detection area of the probe. Future studies should investigate the effects of various parameters and training methods on changes in the PMaj thickness during exercise. These findings enhance our understanding of muscle physiology and the effects of exercise training.

## Conclusions

This study used a deep learning model to comprehensively analyze changes in PMaj thickness during pec-fly exercises using a wearable ultrasound imaging setup. We provide quantitative insights into PMaj thickness alterations in the coronal plane within the entire probe detection region, overcoming the limitations of manual measurements. We found that incorporating RUSI visual biofeedback resulted in augmented PMaj thickness changes, regardless of load intensity. Furthermore, biofeedback has been used to mitigate load-dependent differences, resulting in improved muscle activation strategies. The optimal site for measuring peak PMaj thickness near the rib end was pinpointed. While additional research is needed to fine-tune load intensities and improve segmentation techniques, our streamlined model provides valuable application in fitness training contexts. This study contributes to the understanding of muscle physiology and lays the foundation for exploring various training approaches.

## Data Availability

For access to the experimental data in this paper, please contact the corresponding authors Yongjin Zhou and Yongping Zheng.

## Abbreviations

PMaj: Pectoralis major
RUSI: Real-time ultrasound imaging
1-RM: One-repetition maximum
CNN: Convolutional neural network
RNN: Recurrent neural network
